# Being a Pakistani mother in Catalonia: a mixed methods study

**DOI:** 10.3389/fpsyg.2024.1386029

**Published:** 2024-11-26

**Authors:** Maryam El Khayat, Magda Rivero, M. Teresa Anguera

**Affiliations:** ^1^Department of Cognition, Development and Educational Psychology; Faculty of Psychology, University of Barcelona, Barcelona, Spain; ^2^Faculty of Psychology, Institute of Neurosciences, University of Barcelona, Barcelona, Spain

**Keywords:** parenting, Pakistani migrant families, acculturation, migrant motherhood, polar coordinate analysis, mixed methods

## Abstract

**Introduction:**

In Pakistani migrant families, contextual transformation can affect adult caregivers’ parental skills and their ability to exercise positive parenting. We focused on identifying and describing patterns, practices and beliefs about parenting, identifying differential characteristics between the context of origin and the host context, and exploring Pakistani immigrants’ use of resources or assets in the area of parenting support.

**Methods:**

Participants consisted of 20 women, established in Catalonia, Spain (<5 years of residence) who have children (at least one of preschool-age). We used a method of indirect observation based on verbal narrative data and textual material that allows integration between qualitative and quantitative elements. The analysis of polar coordinate (quantitative) was applied to obtain a map of interrelationships between codes/categories, based on code matrices. This method is innovative as this is the first study we know in which focus groups have been analyzed through polar coordinate.

**Results:**

Our findings underscore the importance of examining in-depth the concept of family, parenting and upbringing from a cross-cultural perspective. Our results suggest that Pakistani migrant mothers dedicate resources and efforts to maintain the values and practices of origin, and to transmit them to the following generations.

**Discussion:**

Constant communication among relatives using new technologies, the desire to return to Pakistan, and the absence of spaces for interaction between native and migrated families facilitate the maintenance of the upbringing model of origin and resistance to change. A nuclear family structure and access to educational and health services promote acculturation processes in favor of adaptation to the new reality.

## Introduction

The concept of parenting refers to the learning, facts, activities or responsibility of the people involved in the care of children. It also refers to their knowledge, attitudes and beliefs about health and nutrition, the importance they attribute to developmental environments, and the learning opportunities that they organize and make available to children within the home (Eraso et al., 2006). The family, as the main context of upbringing, has been one of the main focuses of empirical research in the fields of development and education (Carrión, 2015; Henriquez, 2014; Izzedin-Bouquet and Pachajoa-Londoño, 2009; Torío López et al., 2009). This has allowed us to advance in the knowledge available about the relationships established between fathers, mothers and children, and to determine which contextual and personal factors lead to better development of children (Carrión, 2015; Fuentes-Balderrama et al., 2022; Henriquez, 2014; Izzedin-Bouquet and Pachajoa-Londoño, 2009; Rivero et al., 2023; Vilaseca et al., 2022).

### Parenting, upbringing, and migration

In migrated families there are several contextual transformations derived from the migratory process that can compromise the resources available to them in the exercise of parenting. Settling in a society other than one’s own requires a considerable effort of psychological and cultural adjustment (Berry, 1997; Fuentes-Balderrama et al., 2022; Micolta, 2007), in which various factors are involved. Gallardo (2019) proposes an approach to the subject that considers points in the migration process. In the period before migration, the family’s cultural values should be considered (DeHaan, 2011) and the reasons that led them to emigrate (Aruj, 2009; Buttiler et al., 2023). At the time of migration, it should be observed how the country welcomes the family through its migration policies (Bornstein, 2017). Finally, after migration, the focus should be the loss of references and social support (Ali, 2008; Belsky, 1984; Bornstein and Bohr, 2011), the perception of belonging to a minority group (DeHaan, 2011) and aspects related to the support structure and opportunities provided to the family of migrant origin. Notably, one of the main reasons for migration in these families is the search for better conditions and opportunities for children (Levitt and Glick-Schiller, 2004; Mazzucato, 2008). That is, emphasis is placed on migration as a parental decision (Gallardo, 2019; Perreira et al., 2006).

Within post-migration factors, the concept of parental acculturation has begun to be introduced (Berry et al., 1986; Bornstein and Bohr, 2011; Eltanamly et al., 2022; Gallardo, 2019) related to the dilemmas that parents face when they abandon or retain the typical parenting forms from their culture of origin and adopt or reject the parenting methods of the receiving culture (Gallardo, 2019). This is a particularly sensitive issue, since all cultures have specific parenting patterns that are transmitted across generations, which validate what it means to carry out “successful parenting” (Baker, 2016; Bornstein and Bohr, 2011; Keller et al., 2006; Roer-Stier, 2001). In this way, raising children in a culture different from the initial one creates tension between the expectations of being parents that are validated by the culture of origin and the expectations and demands associated with parental roles in the receiving culture (Roer-Stier, 2001).

In studies of the behavior of parents with their children, it is essential to consider two basic aspects: the cultural principles of the group of which they form a part, which affects how they interpret the needs of their children, and the characteristics of the environment(s) in which they have to raise and educate their children, which modulate their responses to these needs (D’Andrade, 2001; Le Vine, 1980, 2003). The way that parents face difficulties that arise on the path to achieving the goals that have been set will be modulated by the group they belong to and culturally determined mechanisms, in close connection with the context that each society establishes as adaptive. A family that decides to migrate loses the opportunity to act according to its values and traditions. Parents find themselves in the position of choosing between modifying and adapting their practices to the new environment, or maintaining the original practices, even if they are not fully adaptive in the host society (Berry et al., 1986; Deng and Marlowe, 2013; Eltanamly et al., 2022).

### Parenting and upbringing in Pakistan

Recent research conducted in Pakistan has centered on the issue of parenting (Jeong et al., 2019; Obradovic et al., 2016; Rasheed and Yousafzai, 2015; Shamama-Tus-Sabah et al., 2011; Sikander et al., 2018; Yousafzai et al., 2015) and offers us specific knowledge about the defining characteristics of the parenting model at source. In Pakistan, Islam is the official religion and creates a cultural foundation that structures the self and its relationship with immediate family and relatives, and non-family members (Selin, 2014). Therefore, Muslim values provide the core of parenting norms that cut across social and economic classes even though the emphasis and role of religion varies in the lives of families (Zaman, 2014).

Family structure directly affects parenting in Pakistan (Selin, 2014). Families attach enormous importance to duty and obligation, rather than to the individual rights of family members. The most common structure of a family living under the same roof is that of the extended family, vertically and horizontally. This can include three generations: grandparents, sons, their wives, and unmarried sons or daughters and siblings. Usually, the oldest father is the head of the family and its main breadwinner until the adult children can contribute to the family economy. The mother’s responsibility is to manage the household and children, and when they live in a joint family, to care for their in-laws and maintain family harmony.

The upbringing, training and teaching (*tarbyiat*) and education (*taleem*) of children (Erdal et al., 2016) is the responsibility of the parents and members of the extended family, including grandparents, aunts and uncles. Grandmothers are often the key decision-makers on issues related to pregnant mothers and young children in low-resource settings (Chung et al., 2019) and active participants in feeding, bathing and playing with children (Lingam et al., 2014; Sharma and Kanani, 2006). In many contexts, they are perceived as wise and experienced, serving as teachers to young mothers and passing on fundamental traditions and knowledge (Aubel, 2012; Mumtaz and Salway, 2007). According to Moazam (Selin, 2014), the first lesson of childhood is focused on respecting the elders.

### Parenting and upbringing in migrant families of Pakistani origin in Catalonia

Pakistan is among the countries with the most emigrants in the world ranking, with 8.8 million Pakistanis residing abroad. In Europe, Spain is in fourth place among the main receiving countries, just behind the United Kingdom, Italy and Germany.

In Spain, the group from Pakistan has grown continuously in the last decade from 79.984 people in 2002 to 100.496 people in 2022, with a slight population decrease between 2013 and 2016 that coincided with the forecast closure of borders in the United Kingdom, the main European destination, because of the withdrawal of this country from the European Union. More than half of the Pakistani population in Spain, 55.771 people, are in Catalonia (National Institute of Statistics, 2022). However, despite Pakistani being one of the most numerous non-EU populations, little is known about this population in many areas. This is a challenge for professionals responsible for their care and support. The Pakistani population is a very heterogeneous group, in terms of their experiences before the migratory process, and how each one of them has reconstructed their cultural parameters in the host society. The available knowledge about parenting in Pakistani families is still insufficient and not easily transferable to a migration context.

In the present study, carried out using a mixed methods framework (Johnson and Onwuegbuzie, 2004; Karasz and Singelis, 2009), we used a method of indirect observation based on verbal narrative data and textual material (Anguera, 2021; Anguera et al., 2018). This approach is ideally suited for the integration of potentially highly complex qualitative and quantitative elements, especially in situations in which the starting point is the conversations carried out in a focus group. Until recently, the qualitative nature of the material would have disqualified it for use in a mixed methods framework, but recent methodological advances in the diachronic analysis of qualitative data have made its application possible (Anguera, 2020; Anguera et al., 2021; Onwuegbuzie and Jonhson, 2021).

We want to highlight its innovative character in the methodological field as it is the second study that we are aware of in which focus groups are treated with indirect observational methods and analyzed through polar coordinate analysis (Bonilla et al., 2024). Indirect observation is an appropriate method to capture behaviors displayed or reported by participants themselves (in the case of this study, ideas, experiences, expectations and values). Polar coordinate analysis was chosen as it permits analysis of the interrelations between coded behaviors.

As we have seen, migrant families may face various challenges that require psychological and cultural adjustments and can jeopardize successful parenting. These challenges include those related to migration itself, the birth of children, and the parental acculturation process (Berry, 1997; Fuentes-Balderrama et al., 2022; Micolta, 2007). The interaction between the family model and cultural values of the country of origin and the new references of the host society can lead to tensions, opportunities, and challenges that need to be addressed (Baker, 2016; Bornstein and Bohr, 2011; Keller et al., 2006; Roer-Stier, 2001). Moreover, we are aware that the family structure has an impact on parenting in Pakistan (Selin, 2014), with the prevalent extended family arrangement where multiple generations reside under the same roof. After migration, these families may lose their direct support in parenting, daily organization, and the values associated with communal care. Thus, after a review of the literature, we identify several critical topics related to parenting and childrearing among migrant mothers in general and Pakistani mothers in particular that will be addressed in the focus groups. They can be summarized as tensions between parental aims, values and practices in the culture of origin and those of the host culture, and knowledge and use of resources offered by the community.

This study aims to add to the body of knowledge regarding parenting in families of Pakistani origin in the context of migration through its three objectives, which stem from the theoretical and conceptual framework relating to the main topics identified in the scientific literature (1) to offer an approximation to guidelines, practices and beliefs on the upbringing of families of Pakistani origin who migrated to Catalonia; (2) to establish and describe the differential characteristics between the context of origin and the host context that are associated with opportunities and barriers in parenting practices; and (3) to explore the use that these families make of resources or assets in the area related to support in upbringing. The first two objectives highlight the tensions between parental aims, values and practices in the culture of origin and the host culture. The third objective relates to the knowledge and use of the resources offered by the community.

## Method

### Design

Within the mixed methods framework, as we already stated in the introduction section, we used a method of indirect observation based on verbal narrative data and textual material. In this methodological context, our approach to quantification is not based on transforming qualitative data into quantitative ones, as explained in Chang et al. (2009). Instead, we use a more innovative method of quantitizing, QUAL-QUAN-QUAL (Anguera et al., 2020), according to which we organize the qualitative data collected, which we code using the indirect observation instrument. We then apply quality control and data analysis techniques to the code matrices obtained in the registry. This methodology, based on observational procedures, has unique characteristics that make it a mixed method, as discussed in Sánchez-Algarra and Anguera (2013), Anguera et al. (2017), and Anguera and Hernández-Mendo (2016).

The observational design used is nomothetic/punctual/multidimensional (N/P/M) (Anguera et al., 2001). It is nomothetic because the members of each of the three focus groups participate; punctual (although with intra-session follow-up) because each of the focus groups met for one session, which was recorded from start to finish; and multidimensional because, depending on the theoretical framework, various dimensions were proposed as part of the instrument.

### Participants

Participants were 20 mothers between 29 and 49 years old (Mage =35.47, SDage = 4.376) who had between one and five children (Mn_children = 2.37, SDn_children = 1.165) and had been living in Catalonia for a period of between 1 month and 5 years (Myears_cat =3.21, SDyears_cat = 1.512). Each focus group had homogeneous inclusion criteria, but members were heterogeneous in terms of age, schooling, profession, and area of origin (rural or urban).

We selected the participants according to the following inclusion criteria: (1) woman, (2) established in Catalonia, Spain, for no more than 5 years and (3) with at least one child of preschool age, to guarantee that all participants were involved in parenting experiences at the time. The condition of representativeness was not necessary. Data on sociodemographic variables were also collected (Supplementary Table S1).

A maximum of 5 years of residence was established as an inclusion criterion to observe conflicts related to the acculturation process. Five years is the time required to exercise the right to apply for legal permanent residence.

This study was approved by the Bioethics Committee of the University of Barcelona (IRB00003099). Before the meeting, all participants received verbal information in their native language, Urdu, about the objectives of the study, and consent to participate was requested through a document translated into this language. Likewise, we anonymized the transcripts to ensure the confidentiality and privacy of the participants.

### Instruments

We distinguish between observational instruments and recording and analysis instruments.

#### Observation instrument

The indirect observation instrument, which we developed *ad hoc*, combines field format and category systems, based on the theoretical framework and, to a large degree, the responses obtained in the three focus groups (Anguera et al., 2007).

We used a nomothetic approach (Gibbs, 2007) based on grounded theory (Strauss and Corbin, 1990, 1998) in three stages. In the first stage, we fragmented the information that was recorded and transcribed into 500 units of meaning, using complete sentences as a criterion, to which we assigned a descriptor or code. In the second stage, we grouped the codes into categories (axial coding). Finally, in the third stage, we developed themes that expressed the content of each group (selective coding). Once this indirect observation tool had been created, we coded the narratives through the dimensions and category systems. The category systems that were part of the observation instrument were exhaustive and mutually exclusive.

The instrument (Supplementary Table S2) was built by organizing the emerging codes into sub-dimensions and then into six dimensions, aligning with the study’s objectives: Objective1. *Changes after maternity* (CA), *Significance and meaning of maternity* (SE), *Parenting model* (MO), *Basic individual needs of family members* (NE); Objective2. *Acculturation* (AC); and Objective 3. *Support network* (RE). These dimensions emerged from the participants’ responses to the questions that were discussed in the focus groups, following the objectives of the study and supported by the theoretical framework. Each of these dimensions allowed the construction of respective category systems, which met the essential conditions of exhaustivity and mutual exclusivity. Each category was defined, a code was assigned to it, and an example was presented to illustrate that it was extracted from the responses obtained in the focus groups. This observation tool fully meets the requirements outlined in sections 9 and 10 of the MQCOM (Chacón-Moscoso et al., 2019).

We recorded the interviews using a mobile application and an audio recorder (two simultaneous recordings). After each session, we translated and manually transcribed the entire content of the interviews, including the interpreter’s translations during the development of the focus groups.

#### Recording and analysis instruments

We used the Excel program for recording data. Data quality control was performed using the free GSEQ program (Bakeman and Quera, 2011).^1^ Data analysis was conducted using the free program HOISAN (Hernández-Mendo et al., 2012) accessible at www.menpas.com, and the figures were optimized using the free program R (Rodríguez-Medina et al., 2022).^2^

### Procedure

In the planning of the focus groups (Doody et al., 2013; Gozálvez Pérez et al., 2012; Krueger and Casey, 2000), we considered the indications offered by Hamui-Sutton and Varela-Ruiz (2013) concerning the preparation of the focus group, the number of participants, duration, the space, and the role of the moderator.

During October and November 2021, we held meetings with three women of Pakistani origin who are considered community leaders and who participate in the social transformation and inclusion of women from various associations. At these meetings, the women’s collaboration was requested in order to identify and organize three focus groups. From this first contact, the “snowball” technique was used to select the participants (Heckathorn, 2011; Navarrete et al., 2022). This method allows a sample to be defined through references made by people who share the information or know others and who are interested in the research. Participants were contacted through telephone calls and the collaborating institutions were contacted face-to-face.

During November and December 2021, we conducted three focus groups lasting between 73 and 78 min, a duration considered adequate for this purpose (Gerger Swartling, 2006; Krueger and Casey, 2000).

We conducted three focus groups. Each group have been organized independently, both in terms of location and timing, and it had either six or seven participants, following the recommended size of six to twelve participants for focus groups (Gerger Swartling, 2006). The goal was to ensure that everyone had the chance to express themselves, ask questions, and influence the collective results (Webler, 1995). Each group was moderated by a facilitator who asked predetermined questions and guided the discussion to address the research objectives and a professional interpreter of Pakistani origin (both cultural background and gender were considered to facilitate communication and establish a safe space for women). The vehicular language used was Urdu.

The moderator introduced the topics according to a previous script divided into four thematic sections: a common one (A) addressed in the three focus groups, with introductory questions about parenting, and three sections based on the study objectives: questions about guidelines, practices and beliefs regarding upbringing (B); questions aimed at establishing and describing the differential characteristics between the context of origin and the host context that are associated with opportunities and barriers in parenting practices (C); and questions about the use that these families make of resources or assets in the area related to support in upbringing (D). Sections B, C, and D are divided among different focus groups to reduce the number of questions, in line with the recommendations made in previous work on focus groups (Gerger Swartling, 2006; Krueger and Casey, 2000). We assigned sections to each focus group based on the order of the sections and the timing of the group, ensuring that each section was covered in two groups (with the exception of section A, which was introductory and was included in all groups).

We distributed the questions among the groups according to the following organization:

- Focus group 1: sections A, B and C.
- Focus group 2: sections A, C and D.
- Focus group 3: sections A, B and D.

The interviews were audio-recorded, translated and transcribed in full, including the interpreter’s translations during the development of the focus groups.

To code the answers obtained in the focus groups, we segmented the code using syntactic criteria (Anguera, 2020; Anguera, 2021; Bonilla et al., 2024) to provide the textual units. Once all textual units were available, the recording was completed using the Excel program, which involved creating a matrix code with six columns corresponding to the dimensions of the indirect observation instrument and as many rows as textual units obtained. Finally, the corresponding code was placed in each box. This was the last action that corresponded to the first stage “WHAT” in the *mixed methods approach*.

Based on the code matrix obtained, data quality control was conducted, followed by data analysis as specified in the subsequent sections.

### Data quality control

The analysis of concordance of the coded record between two independent observers (coding 33% of the total textual units), with which the QUAN stage of the QUAL-QUAN-QUAL mixed methods approach begins, obtained a Kappa coefficient (Cohen, 1960) of 0.7999. We rated this as satisfactory (Landis and Koch, 1997).

### Data analysis strategy

On the quantitative side, the analysis of polar coordinate was used (Sackett, 1980) to obtain a map of interrelationships between the codes/categories based on the code matrices to which we have referred (Anguera et al., 2021). The polar coordinate analysis is extremely strong and is based on the quantitative study of the sequentiality of the qualitative records that are obtained (successive interventions by the participants in the focus groups).

This requires initial designation of the focal behavior (the category that, according to the goals of the study, is proposed as the center to analyze its interrelationships with the other categories), the conditioned behaviors (all behaviors about which we want to know their interrelationship with the focal behavior), and the number of lags (in a sequential recording, the lag is the distance, measured in number of codes, between the criterion behavior and the conditioned behavior), both prospective (positive) and retrospective (negative), that will be considered (in our case, the number ranged from +1 to +5, and from −1 to −5 respectively).

The polar coordinate analysis is considered a second stage of the lag sequential analysis (Bakeman, 1978). In the polar coordinate analysis, the prospective perspective (from the moment a focal behavior occurs) and the retrospective perspective (textual units that happened before the occurrence of the focal behavior) are integrated. However, we are not referring to the classic retrospective approach (Sackett, 1980), but to genuine retrospectively as proposed by Anguera (1997), since it is consolidated and incorporated into the HOISAN program (Hernández-Mendo et al., 2012) to which we will refer.

The large volume of calculations that would be required to develop prospective and retrospective approaches is greatly simplified by the parameters Z _sum prospective_ and Z _sum retrospective_. Sackett (1980) proposed these parameters from a proposal made by Cochran (1954) to reduce data, as long as they are independent. Here they are independent because they are the successive results obtained in lag sequential analyses that have the focal behavior of the polar coordinate analysis as the criterion behavior, the same conditioned behaviors and successive lags (whether prospective or retrospective).

Based on prospective Z_sum_ and retrospective Z_sum values_, Sackett (1980) proposed the vectorization of the relationships between focal behavior and conditioned behaviors. There will be as many vectors as conditioned behaviors. Each vector has a length or radius Length=$\sqrt{{\left(\text{Zsum}\;\text{prospective}\right)}^{2}+{\left(\text{Zsum}\;\text{retrospective}\right)}^{2}}$, and the angle *φ* of the vector is found from its trigonometric function Arc sin, which is expressed as follows: Arc sin φ=$\frac{\left(\text{Zsum}\;\text{retrospective}\right)}{\text{Length}}\text{.}$

This angle φ will be subject to adjustments depending on the quadrant it is in, as indicated in Table 1 (Anguera et al., 2021). In addition, this table provides the indications for the interpretation of the vector. It shows the relationship between the focal behavior and the respective conditioned behavior (each conditioned behavior generates a vector), and it is reciprocal.

**Table 1:** Profile of the vectors according to the quadrant in which they are found.

Quadrant	Angle	Relationships between focal behavior and conditioned behaviors
I	(0 < *φ* < 90) = *φ*	Focal behavior and conditioned behavior activate each other.
II	(90 < φ < 180) = 180-φ	Focal behavior inhibits conditioned behavior, which activates focal behavior.
III	(180 < φ < 270) = 180 + φ	Focal behavior and conditioned behavior mutually inhibit each other.
IV	(270 < φ < 360) = 360-φ	Focal behavior activates conditioned behavior, which inhibits focal behavior.

The polar coordinate analysis was carried out using the free program HOISAN (Hernández-Mendo et al., 2012), which provides, for each conditioned behavior, the quadrant in which the vector is located, Z _sum prospective_, Z _sum retrospective_, the vector length or radius, the significance level, and the angle. The graphic representation has been optimized using R (Rodríguez-Medina et al., 2022).

The great power and applicability of polar coordinate analysis have become evident in recent years in different areas of behavior, such as Developmental Psychology (Belza et al., 2020; Sagastui et al., 2021; Sagastui et al., 2023), Educational Psychology (Alarcón-Espinoza et al., 2023; García-Fariña et al., 2021; Terroba et al., 2022), Clinical Psychology (Arias-Pujol et al., 2022; Rodríguez-Medina et al., 2022), Health Psychology (Muñoz-Violant et al., 2021; Portell et al., 2019), Sports Psychology (Aragon et al., 2017; Iván-Baragaño et al., 2023; Izquierdo and Anguera, 2021; Maneiro et al., 2019) and Environmental Psychology (Pérez-Tejera et al., 2022), among others (Chacón-Moscoso et al., 2023; Del Giacco et al., 2020). It has been found to be a strong technique that greatly reduces data while it provides reliable information on the interrelationships between the categories of the observation instrument and specifically between the proposals, such as the focal behavior and conditioned behaviors.

## Results

Our interest is focused on identifying and describing patterns, practices and beliefs about parenting in families of Pakistani origin and identifying differential characteristics between the context of origin and the host context that are associated with opportunities and barriers in parenting practices. Our aim was also to determine the use that Pakistani families make of the resources or assets in the area of parenting support.

In this section, we describe the results of detecting regularities between emergent codes and their grouping into higher-order categories or central categories. The following are the results of the polar coordinate analysis of the significant relationship of focal and conditioned behaviors that activate each other (quadrant I) in the selected units. The tables have been divided into three groups that correspond to the objectives of the study.

To thoroughly study the relationship between focal behaviors and all other conditioned behaviors, we performed 81 polar coordinate analyses considering each category as a focal behavior and all other behaviors shown sequentially as conditioned ones (included in the Supplementary material). We selected six focal behaviors, one for each coded dimension, based on the study objectives, their connection with the theoretical framework, and considering that the interrelationships found with each conditioned behavior are highly significant.

### Polar coordinate analysis

For all tables (Tables 2–7), the prospective and retrospective Zsum values are presented in the second and third columns, and the values of vector length and its angle are shown in the fourth and fifth columns.

**Table 2:** Significant and highly significant vectors corresponding to quadrant I focal behavior: CA1 organization.

Category	Quadrant	Prospective Zsum	Zsum Retrospective	Ratio	Length	Significance	Angle
CA_CA21	I	2.03	4.81	0.92	5.22	**	67.13
CA_CA3	I	4.85	1.89	0.36	5.2	**	21.26
CA_CA42	I	4.82	7.6	0.84	9	**	57.63
MO_MO11	I	5.59	4.44	0.62	7.14	**	38.47
MO_MO12	I	6.21	6.21	0.71	8.78	**	45.02
MO_MO22	I	7.32	3.87	0.47	8.27	**	27.85
AC_AC222	I	3.21	0.56	0.17	3.26	**	9.86
AC_AC232	I	2.68	0.8	0.29	2.8	**	16.56
AC_AC233	I	7.04	1.34	0.19	7.17	**	10.81
AC_AC241	I	3.42	2.03	0.51	3.98	**	30.68
RE_RE112	I	0.06	5.31	1	5.31	**	89.32

Focal behaviors and conditioned behaviors activate each other.

**Table 3:** Significant and highly significant vectors corresponding to quadrant I focal behavior: SE21 responsibility.

Category	Quadrant	Prospective Zsum	Zsum Retrospective	Ratio	Length	Significance	Angle
MO_MO411	I	0.06	3.77	1	3.78	**	89.09
MO_MO414	I	18.01	21.83	0.77	28.3	**	50.48
MO_MO425	I	6.53	1.88	0.28	6.79	**	16.06
NE_NE11	I	1.02	4.31	0.97	4.43	**	76.73
NE_NE13	I	0.57	5.96	1	5.98	**	84.51
RE_RE321	I	0.73	3.45	0.98	3.53	**	78.04
RE_RE322	I	1.29	3.11	0.92	3.37	**	67.5

Focal behaviors and conditioned behaviors activate each other.

**Table 4:** Significant and highly significant vectors corresponding to quadrant I focal behavior: MO2111 present family values.

Category	Quadrant	Prospective Zsum	Zsum Retrospective	Ratio	Length	Significance	Angle
MO_MO32	I	0.58	5.98	1	6	**	84.44
MO_MO5	I	4.63	5.98	0.79	7.56	**	52.26
NE_NE23	I	2.28	2.28	0.71	3.22	**	44.97
AC_AC211	I	2.98	2.98	0.71	4.21	**	44.99
AC_AC213	I	5.73	10.5	0.88	11.96	**	61.39
AC_AC223	I	2.98	1.71	0.5	3.43	**	29.87
AC_AC245	I	13.87	9.77	0.58	16.96	**	35.16
AC_AC4	I	3.26	6.71	0.9	7.46	**	64.06

Focal behaviors and conditioned behaviors activate each other.

**Table 5:** Significant and highly significant vectors corresponding to quadrant I focal behavior: NE21 link.

Category	Quadrant	Prospective Zsum	Zsum Retrospective	Ratio	Length	Significance	Angle
MO_MO11	I	2.51	1.83	0.59	3.1	**	36.1
MO_MO31	I	9.61	4.94	0.46	10.81	**	27.2
MO_MO32	I	0.58	10.31	1	10.33	**	86.78
MO_MO415	I	4.9	4.91	0.71	6.93	**	45.03
NE_NE11	I	2.67	1.01	0.35	2.85	**	20.76
NE_NE21	I	11.07	11.07	0.71	15.66	**	4. 5
NE_NE23	I	1.34	2.75	0.9	3.06	**	64.09
AC_AC211	I	4.93	0.27	0.06	4.94	**	3.16
AC_AC231	I	7.6	1.02	0.13	7.67	**	7.61
AC_AC234	I	3.26	4.6	0.82	5.64	**	54.73
AC_AC241	I	5.96	1.01	0.17	6.04	**	9.66

Focal behaviors and conditioned behaviors activate each other.

**Table 6:** Significant and highly significant vectors corresponding to quadrant I focal behavior: AC4.

Category	Quadrant	Prospective Zsum	Zsum Retrospective	Ratio	Length	Significance	Angle
MO_MO2112	I	8.76	7.45	0.65	11.5	**	40.4
MO_MO32	I	2.96	0.01	0	2.96	**	0.27
MO_MO411	I	4.43	1.95	0.4	4.84	**	23.8
MO_MO413	I	6.66	4.73	0.58	8.17	**	35.35
MO_MO5	I	3.69	1.48	0.37	3.98	**	21.89
AC_AC211	I	2.78	5.7	0.9	6.34	**	63.95
AC_AC213	I	6.58	3.11	0.43	7.28	**	25.32
AC_AC223	I	2.57	3.95	0.84	4.72	**	56.95
AC_AC236	I	1.39	5.26	0.97	5.44	**	75.21
AC_AC245	I	1.29	3.52	0.94	3.75	**	69.92
AC_AC4	I	14.71	14.71	0.71	20.8	**	4.5

Concern for the loss of cultural/religious values and/or acquisition of practices of the host society. Focal behaviors and conditioned behaviors activate each other.

**Table 7:** Significant and highly significant vectors corresponding to quadrant I focal behavior: RE3311 barriers to participation.

Category	Quadrant	Prospective Zsum	Zsum Retrospective	Ratio	Length	Significance	Angle
MO_MO416	I	5.49	1.48	0.26	5.69	*	15.04
NE_NE12	I	6.1	2.81	0.42	6.72	**	24.76
RE_RE111	I	0.25	3.72	1	3.73	**	86.21
RE_RE311	I	2.12	0.55	0.25	2.19	**	14.5
RE_RE3311	I	17.83	17.83	0.71	25.21	*	4.5
RE_RE3312	I	11	11.01	0.71	15.56	**	45.01
RE_RE332	I	4.94	2.1	0.39	5.36	**	23

Focal behaviors and conditioned behaviors activate each other.

**Figure 1:**
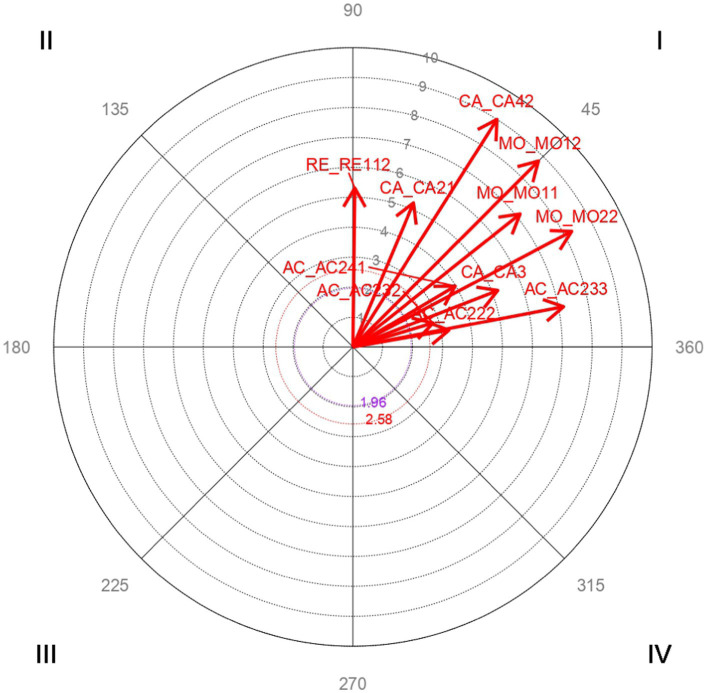
Analysis of polar coordinate of focal behaviorCA1 organization quadrant I. The vector maps show the relationships between the focal behavior and the rest of the conditioned behaviors. Vectors in quadrant I have a positive prospective and retrospective Zsum. Vectors in quadrant II have a positive retrospective and negative prospective Zsum. Vectors in quadrant III have a negative prospective and retrospective Zsum. Vectors in quadrant IV have a positive prospective Zsum and a negative retrospective Zsum. Significant and highly significant relationship vectors (length > 1.96, *p* < 0.05 and length > 2.58, *p* < 0.01, respectively) are plotted.

**Figure 2:**
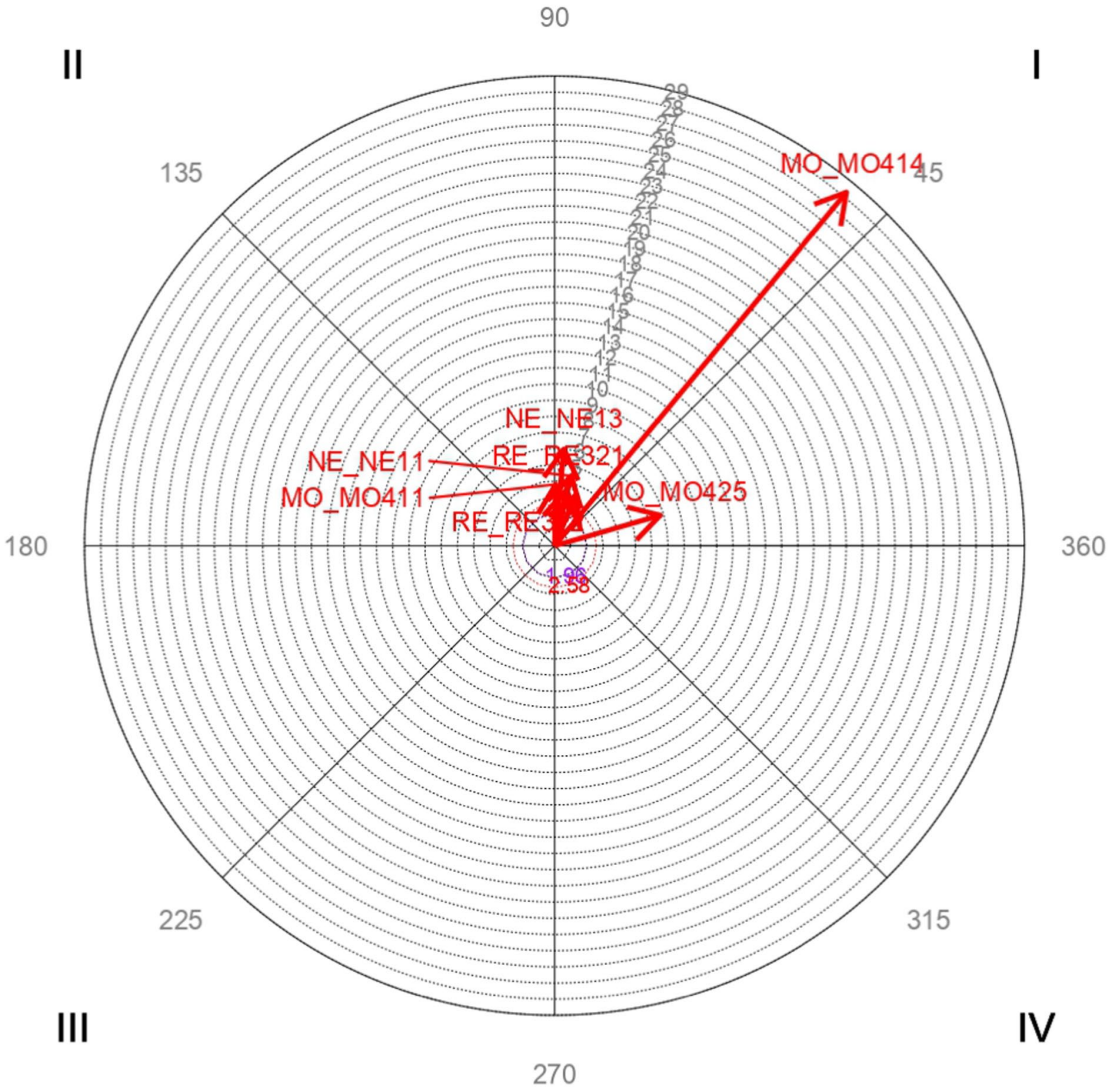
Analysis of polar coordinate of focal behavior SE21 responsibility quadrant I. The vector maps show the relationships between the focal behavior and the rest of the conditioned behavior. Vectors in quadrant I have a positive prospective and retrospective Zsum. Vectors in quadrant II have a positive retrospective and negative prospective Zsum. Vectors in quadrant III have a negative prospective and retrospective Zsum. Vectors in quadrant IV have a positive prospective Zsum and a negative retrospective Zsum. Significant and highly significant relationship vectors (length > 1.96, *p* < 0.05 and length > 2.58, *p* < 0.01, respectively) are plotted.

**Figure 3:**
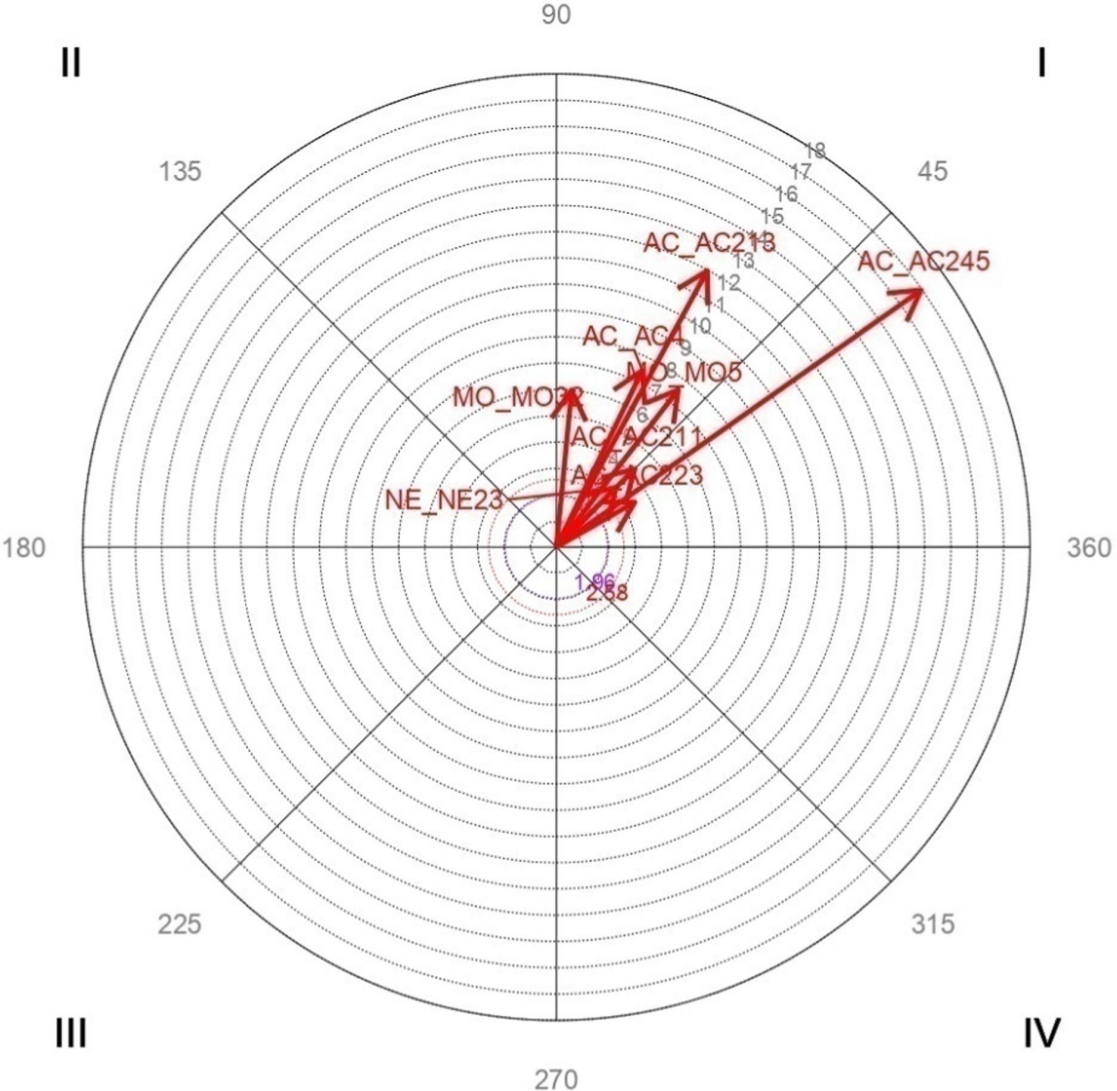
Analysis of polar coordinate of focal behavior MO2111 present family values quadrant I. The vector maps show the relationships between the focal behavior and the rest of the conditioned behaviors. Vectors in quadrant I have a positive prospective and retrospective Zsum. Vectors in quadrant II have a positive retrospective and negative prospective Zsum. Vectors in quadrant III have a negative prospective and retrospective Zsum. Vectors in quadrant IV have a positive prospective Zsum and a negative retrospective Zsum. Significant and highly significant relationship vectors (length > 1.96, *p* < 0.05 and length > 2.58, *p* < 0.01, respectively) are plotted.

**Figure 4:**
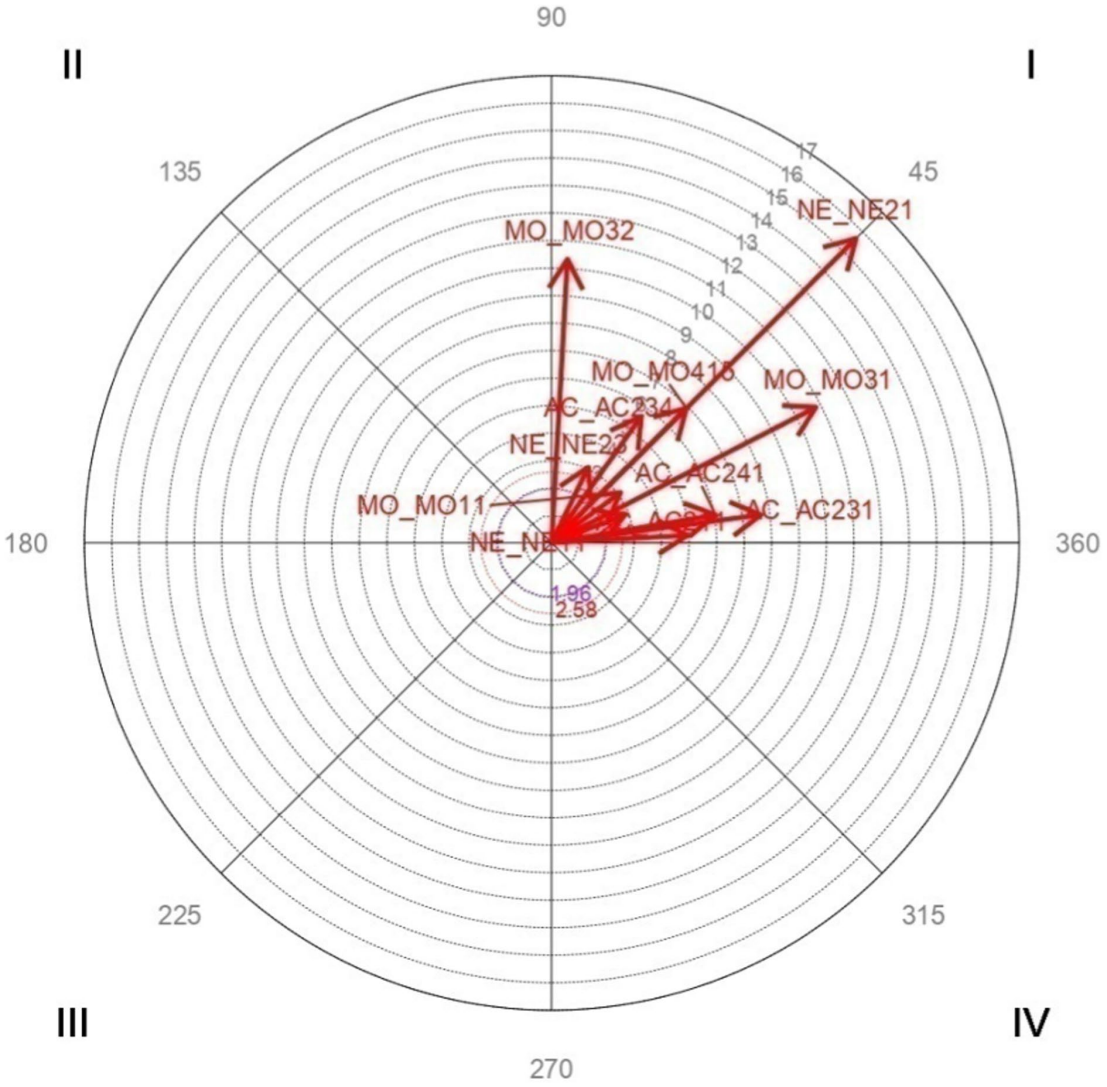
Analysis of polar coordinate of focal behavior NE21 link quadrant I. The vector maps show the relationships between the focal behavior and the rest of the conditioned behaviors. Vectors in quadrant I have a positive prospective and retrospective Zsum. Vectors in quadrant II have a positive retrospective and negative prospective Zsum. Vectors in quadrant III have a negative prospective and retrospective Zsum. Vectors in quadrant IV have a positive prospective Zsum and a negative retrospective Zsum. Significant and highly significant relationship vectors (length > 1.96, *p* < 0.05 and length > 2.58, *p* < 0.01, respectively) are plotted.

**Figure 5:**
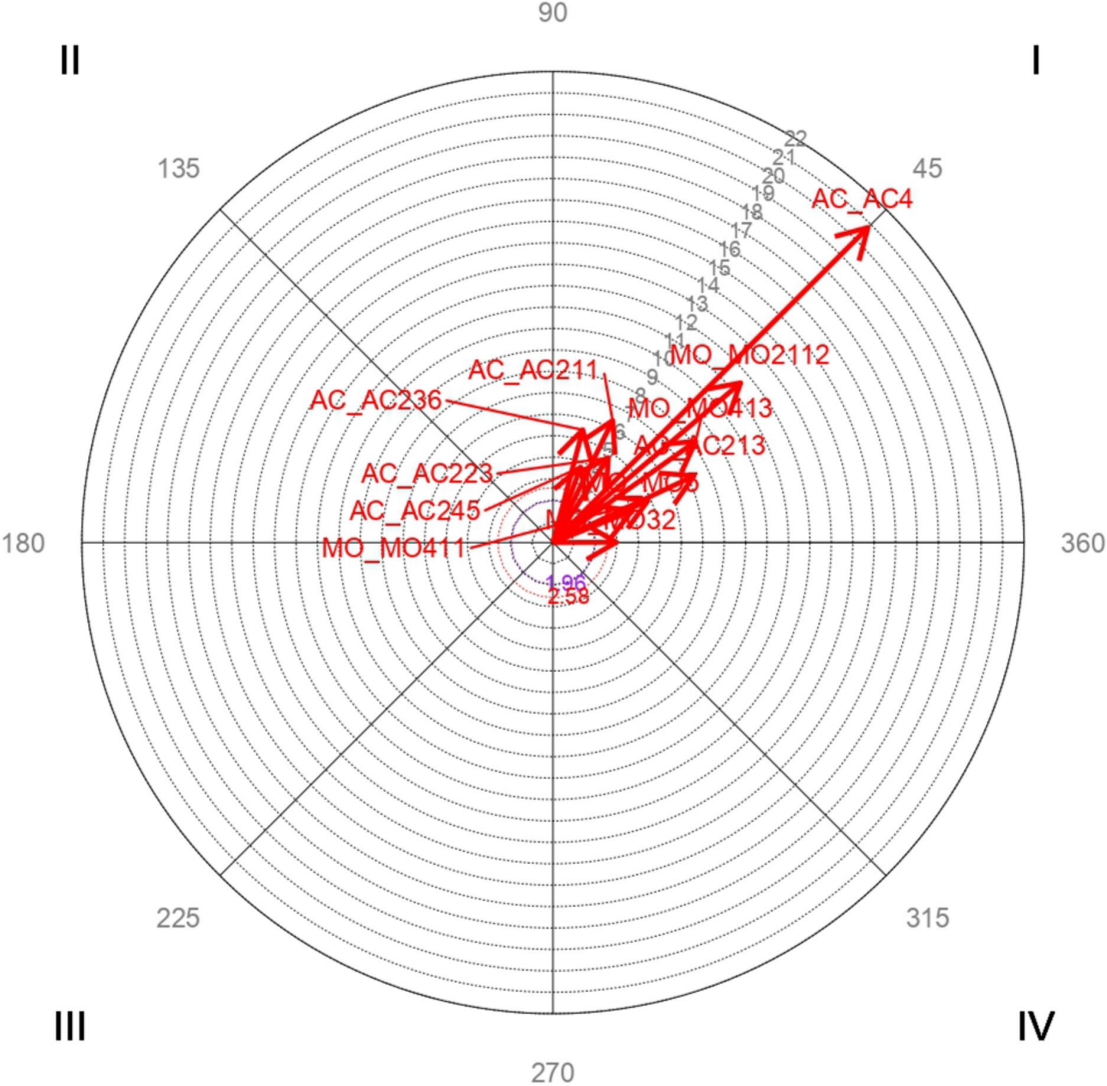
Analysis of polar coordinate of focal behavior AC4 concern for the loss. Concern for the loss of cultural/religious values and/or acquisition of practices of the host society quadrant I. The vector maps show the relationships between the focal behavior and the rest of the conditioned behaviors. Vectors in quadrant I have a positive prospective and retrospective Zsum. Vectors in quadrant II have a positive retrospective and negative prospective Zsum. Vectors in quadrant III have a negative prospective and retrospective Zsum. Vectors in quadrant IV have a positive prospective Zsum and a negative retrospective Zsum. Significant and highly significant relationship vectors (length > 1.96, *p* < 0.05 and length > 2.58, *p* < 0.01, respectively) are plotted.

**Figure 6:**
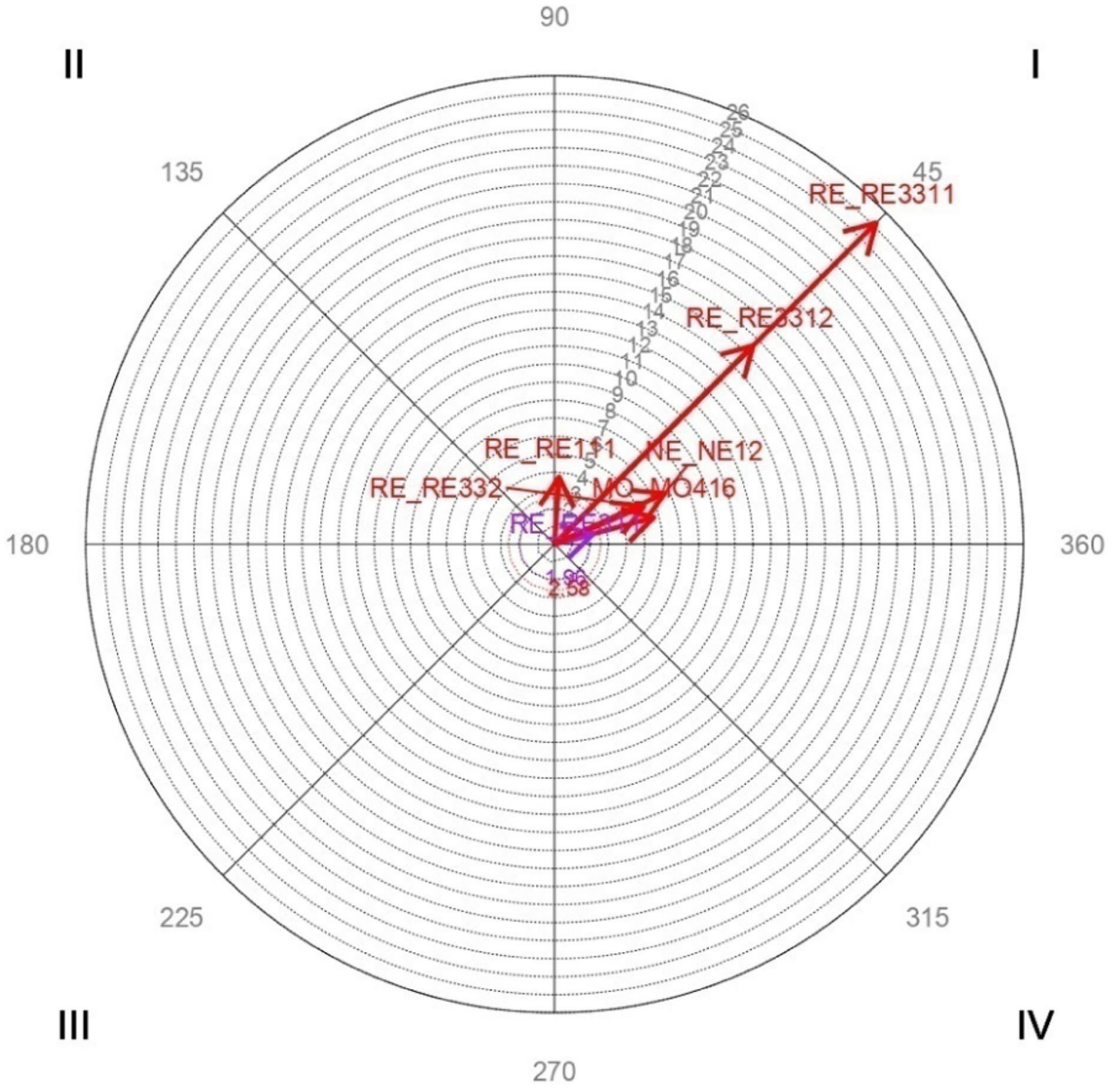
Analysis of polar coordinate of focal behavior RE3311 barriers to participation quadrant I. The vector maps show the relationships between the focal behavior and the rest of the conditioned behaviors. Vectors in quadrant I have a positive prospective and retrospective Zsum. Vectors in quadrant II have a positive retrospective and negative prospective Zsum. Vectors in quadrant III have a negative prospective and retrospective Zsum. Vectors in quadrant IV have a positive prospective Zsum and a negative retrospective Zsum. Significant and highly significant relationship vectors (length > 1.96, *p* < 0.05 and length > 2.58, *p* < 0.01, respectively) are plotted.

Vector maps (Figures 1–6) are presented through polar coordinate analysis, to provide a map of interrelationships between the designated focal and conditioned behaviors.

### Guidelines, practices, and beliefs about parenting in families of Pakistani origin

Table 2 highly significant and mutually activating interactions (quadrant I) were obtained between the focal behavior *CA1 Organization* and the conditioned behaviors *CA21 Changes in the focus of conversation* with a radius of length 5.22 and an angle of 67.7°; *CA3 Tension in the distribution of time between husband and child* with a radius of length 5.2 and an angle of 21.26°; *CA42 Loss of freedom* with a radius of length 9 and an angle of 57.63°; *MO11 Support of the family-in-law in bringing up their children* with a radius of length 7.14 and an angle of 38.47°; *MO12 Responsibilities with the extended family make parenting difficult* with a radius of length 8.78 and an angle of 45.02°; *MO22 Difficulties due to the loss of extended family in support for bringing up the children* with a radius of length 8.27 and an angle of 27.85°; *AC222 Greater difficulty in work-life balance* with a radius of length 3.26 and an angle of 9.86°; *AC232 Cultural and linguistic differences hinder social inclusion* with a radius of length 2.8 and an angle of 16.56°; *AC233 Ease of work-life balance* with a radius of length 7.17 and an angle of 10.81°; *AC241 Other responsibilities make it difficult to dedicate time to parenting* with a radius of length 3.98 and an angle of 30.98°; *RE112 Information between equals* with a radius of length 5.31 and an angle of 89.32°.

Figure 1 shows the highly significant vectors for the focal behavior CA1 Organization and the 11 conditioned behaviors.

Table 3 highly significant and mutually activating interactions (quadrant I) were found between the focal behavior *SE21 Responsibility* and the conditioned behaviors *MO411 Transmission of cultural values, practices and attitudes* with a radius of length 3.78 and an angle of 89.091°; *MO414 Support in studies* with a radius of length 28.3 and an angle of 50.48°; *MO425 Tasks that require linguistic competence* with a radius of length 6.79 and an angle of 16.06°; *NE11 Emotional well-being of the mother* with a longitude radius of 4.43 and an angle of 76.73°; *NE13 Physical well-being of the mother* with a radius of length 5.98 and an angle of 84.51°; *RE321 Opportunities of the educational system* with a radius of length 3.53 and an angle of 78.04°; and *RE322 Limitations of the educational system* with a radius of length 3.37 and an angle of 67.5°.

Figure 2 shows the highly significant vectors for the focal behavior *SE21 responsibility* and the 7 conditioned behaviors.

Table 4 highly significant and mutually activating interactions (quadrant I) were obtained between the focal behavior *MO2111 Present family values* and the conditioned behaviors *MO32 Religion* with a radius of length 6 and an angle of 84.44°; *MO5 Culturally determined practices* with a radius of length 7.56 and an angle of 52.26°; *NE23 Home environment* with a radius of length 3.22 and an angle of 44.97°; *AC211 More dedication of time and/or a better link* with a radius of length 4.21 and an angle of 44.99°; *AC213 It improves the transmission of cultural values and practices* with a radius of length 11.96 and an angle of 61.39°; *AC223 Cultural and linguistic differences hinder social inclusion* with a radius of length 3.43 and an angle of 29.87°; *AC245 Difficulty controlling habits and behaviors of children* with a radius of length 16.96 and an angle of 35.16°; *AC4 Concern for the loss of cultural/religious values and/or acquisition of practices of the host society* with a radius of length 7.46 and an angle of 64.06°.

Figure 3 shows the highly significant vectors for the focal behavior MO2111 *Present Family Values* and the 8 conditioned behaviors.

Table 5 highly significant and mutually activating interactions(quadrant I) were obtained between the focal behavior *NE21 Link* and the conditioned behaviors MO11 *Support of the family in-law in parenting* with a radius of length 3.1 and an angle of 36.1°; MO31 *Personal autonomy* with a radius of length 10.81 and an angle of 27.2°; MO32 *Religion* with a radius of length 10.33 and an angle of 86.78°; MO415 *Emotional support and link* with a radius of length 6.93 and an angle of 45.03°; NE11 *Emotional well-being* with a length radius of 2.85 and an angle of 20.76°; NE23 *Home environment* with a radius of length 3.06 and an angle of 64.09°; AC211 *More dedication of time and/or a better link* with a radius of length 4.94 and an angle of 3.16°; AC231 *Family-in-law link* with a radius of length 7.67 and an angle of 7.61°; AC234 *More options to occupy your free time* with a radius of length 5.64 and an angle of 54.73°; AC241 *Other responsibilities make it difficult to dedicate time to parenting* with a radius of length 6.04 and an angle of 9.66°.

Figure 4 shows the highly significant vectors for the focal behavior NE21 *Link* and the 11 conditioned behaviors.

### Differential characteristics between the context of origin and the host context that is associated with opportunities and barriers in parenting practices

Table 6 highly significant and mutually activating interactions (quadrant I) were obtained between the focal behavior *AC4 Concern for the loss of cultural/religious values and/or acquisition of practices of the host society* and the conditioned behaviors *MO2112 Loss of values after migration* with a radius of length 11.5 and an angle of 40.4°; *MO32 Religion* with a radius of length 2.96 and an angle of 0.27°; *MO411 Transmission of cultural values, practices and attitudes* with a radius of length 4.84 and an angle of 23.8°; *MO413 Surveillance* with a radius of length 8.17 and an angle of 35.35°; *MO5 Culturally determined practices* with a radius of length 3.98 and an angle of 21.89°; *AC211 Longer dedication time and better bond* with a radius of length 6.34 and an angle of 63.95°; *AC213 It improves the transmission of cultural values and practices* with a length radius of 7.28 and an angle of 25.32°; *AC223 Cultural and linguistic differences hinder social inclusion* with a radius of longitude 4.72 and an angle of 56.95°; *AC236 To share cultural values and practices* with a radius of length 5.44 and an angle of 75.21°; *AC245 Difficulty controlling habits and behaviors of children* with a radius of length 3.75 and an angle of 69.92°.

Figure 5 shows the highly significant vectors for the focal behavior *AC4 Concern for the loss of cultural/religious values and/or acquisition of practices of the host society* and the 11 conditioned behaviors.

### Determine the use they make of resources or assets in the area of parenting support

Table 7 highly significant and mutually activating interactions (quadrant I) were obtained between the focal behavior *RE3311 Barriers to participation* and the conditioned behaviors *MO416 Husband care* with a radius of length 5.69 and an angle of 15.04°; *NE12 Family support* with a radius of length 6.72 and an angle of 24.76°; *RE111 Information between equals* with a radius of length 3.73 and an angle of 86.21°; *RE311 Opportunities offered by the health system* with a radius of length 2.19 and an angle of 14.5°; *RE3312 Opportunities to participate* with a radius of length 15.56 and an angle of 45.01°; *RE332 No need or interest in participating* with a radius of length 5.36 and an angle of 23°.

Figure 6 shows the significant and highly significant vectors for the focal behavior *RE3311 Barriers to participation* and the 7 conditioned behaviors.

## Discussion

We interpret and describe the significance of our findings in three sections according to the study objectives.

### Patterns, practices, and beliefs about parenting in families originally from Pakistan

A first point to highlight refers to the *organization* (CA21) dimension, defined in terms of the changes that occur after maternity in the timing of activities, priorities and new tasks that are added to the usual responsibilities of caring for the family and the home (Table 2). This reveals changes in routines and schedules to adapt to the new needs and rhythms of the children in the family and the incorporation of new tasks related to upbringing. This new organization causes interpersonal conflicts in the couple (husbands feel displaced and receive little attention to their needs) (CA3), and intrapersonal conflicts (mothers see their freedom and personal time reduced)(CA42). The focus of the family’s conversation is changed (CA21), with greater attention to aspects related to caring for the baby.

According to the concept of parenting defined by Eraso et al. (2006), within the family, Pakistani mothers assume responsibility for care (SE21) (Table 3) (Goodnow, 1985; Henriquez, 2014; Torío López et al., 2009). This care includes management of the home (cleanliness and order, food and clothing); education and transmission of values, cultural and religious practices (MO411) (Goodnow, 1985); the care of mother–child, family and community ties; monitoring formal education and stimulating the intellect and competencies of children, especially regarding their autonomy (MO414) (Deci and Ryan, 1987; Frodi et al., 1985; Landry et al., 1997).

Cultural (MO2111) and religious (MO32) values are fundamental in the maternal role and the assumption of related responsibilities (Table 4). Motherhood is conceived as a divine mandate and a life goal, so society and the family prepare women for it. In contrast, the paternal role is mainly focused on economic support (MO421). Fathers are responsible for procedures that require linguistic competence outside the home (medical visits, family-school relationships, etc.) (MO425). They play a secondary role in upbringing and participate mainly in moments of leisure and mediated monitoring of the education of their children (MO428).

Mothers assume the responsibility for transmitting cultural guidelines and practices (AC213), monitoring, reviewing and redirecting behaviors that are contrary to their own and facilitating the children’s participation in community and religious spaces (MO32). In general, a desire to maintain the values and cultural practices of origin in the children of the family is present. The mothers expressed fear of the new generation’s assimilation into the receiving society and the incorporation of associated behaviors that are contrary to the values in the country of origin, especially during adolescence (D’Andrade, 2001; Le Vine, 1980, 2003) (AC4). Likewise, they had some difficulties in correctly supporting their children’s learning and schooling due to linguistic, cultural, knowledge and value differences between educational systems (AC223). These factors generated a desire to return to their country of origin(AC3) (Erdal et al., 2016)

As notable aspects in the assessment of parenting in Pakistan and Catalonia, mothers expressed a longing for living with family-in-law in Pakistan, the ties that are associated with this, and the facilities offered in terms of organizational support and distribution of tasks (Ali, 2008; Belsky, 1984; Bornstein and Bohr, 2011) (MO11). However, they positively valued the possibility of spending time raising and caring for their children in the new context, free from taking on other family responsibilities focused on caring for the group (MO12).

One point to consider is the basic needs that mothers identified as variables that determine the context of the development of their children and the possibility of carrying out successful parenting (Table 5). The emotional (NE11) and physical (NE13) well-being of the mother was mentioned as a personal variable that generates better development. In addition, a conflict-free environment (NE23) in the context of the home and the need to allocate time and space to meet the emotional and material needs of the children (AC211) were mentioned as aspects that relate to a better mother–child relationship (Ainsworth, 1973; Bowlby, 1958; Rodrigo and Palacios, 1998).

### Differential characteristics between the context of origin and the host context that is associated with opportunities and barriers in parenting practices

The loss of family, culture (Delgado-Gaitan and Trueba, 1991), religious and identity values of origin and the incorporation of host practices and the values of the host society into the new generations was a source of family concern (AC4) (Table 6) (Bolognani, 2014; Bornstein and Bohr, 2011; Eltanamly et al., 2022; Foner and Dreby, 2011; Gallardo, 2019; Giguère et al., 2010). That is why families devote time to monitoring habits, behaviors and practices (MO413) (Musitu et al., 1988; Shaw, 2000) and the informal teaching (MO411) of religion from an early age at home and in places of worship (MO32). Attending a community mosque offers mothers a place for learning and socializing among equals (AC213). The family-in-law of origin continues to maintain a certain control over the migrated family and expects adequate continuity of family values from the latter. Interestingly, no longer living with family-in-law, a situation that is inherent to the migration process, is the reason for the loss of one of the most precious values: the collectivist conception of the individual, associated with dedication to the group and the community(MO2112), in favor of individuality and personal freedom (Eltanamly et al., 2022; Selin, 2014).

Regarding the acculturation process itself, which is understood as the modification of cognition and behaviors as a result of contact with different cultural groups (Berry, 1997; Berry et al., 1986; Szapocznik et al., 1978), the study participants stated that they had little interaction with native families and few common spaces for participation. They perceived that Catalan mothers had little willingness to share knowledge. The language barrier and cultural differences were perceived as a barrier to their social inclusion and participation (AC223). Despite this, there was widespread appreciation among participants for good parenting practices in native Catalan families (AC122). The ability to set limits and the time spent interacting with children stood out as strengths. The Pakistani mothers perceived that these aspects need to be improved by women in their community raising their children (AC124). In contrast, they observed that Catalan women dedicate less time to family care (AC123). They associated this time particularly with cooking. For Pakistani migrant mothers, spending time cooking is a sign of affection and care for the family.

### Determining the use they make of the resources or assets in the area of parenting support

Some barriers reduce mothers’ participation in local parenting spaces and in family activities offered in their area, in which they do not feel addressed (Table 7). They usually do not access information about these spaces and if they do, they do not consider that the spaces meet their needs (RE3311). Usually, mothers convey a feeling of loneliness in raising children, (AC231) a lack of space in which they can share and learn from each other and from professionals with a cross-cultural perspective who can accompany them according to their idiosyncrasies. Participation in organizations is concentrated mainly in religious spaces (RE111).

The relatives, from a distance, are presented as a source of information and constant validation (RE211). The use of information and communication technologies (ICT) has facilitated fluid communication between the relatives in Pakistan and the nuclear family in Catalonia and the possibility of carrying out, to a certain extent, shared upbringing between the contexts (Bornstein and Bohr, 2011; Keller et al., 2006; Roer-Stier, 2001). The support network in Catalonia is based on the relationship with other migrant mothers in the community who offer, sporadically, emotional and logistic support (RE112) and the possibility of sharing experiences and information about parenting (RE111).

Regarding public resources in Catalonia, social equity in access to quality services and good treatment was valued positively (RE311), in contrast to the country of origin. The health system stood out as a benchmark in monitoring and follow-up during pregnancy and childbirth and for facilitating access to information and care in situations of difficulty or complication. Regarding parenting indications and guidelines that are offered, those that do not clash with culturally determined values and practices are accepted and incorporated (AC112). As an example, the difficulties in following instructions received about the cultural practice of positioning the baby that gives rise to *flat head syndrome* or *positional plagiocephaly* are highlighted. The participants express discrepancies with the professionals when they positively assess their practice, or they avoid a possible conflict within the framework of the family-in-law in the country of origin.

The educational system was highly valued for offering global, holistic attention to children, better emotional education and for having a greater capacity to detect and attend to difficulties and barriers in learning (RE321). However, a perceived low educational level in each primary school stage was considered a barrier to a possible return to the country of origin (RE322): particularly mothers expressed concerns about the low English level and the excess of playing time during the early years (RE323).

## Conclusion

Our findings underscore the importance of examining the concepts of family, parenting, and upbringing from a cross-cultural perspective, to obtain knowledge that helps the design of interventions that are adapted to the real needs of migrant families. To the best of our knowledge, this is the first study to explore concurrent patterns and inter-relational code maps between focal behaviors related to the parenting model, acculturation traits, characteristics of the support network and the use of local resources for support in maternity and parenting. It is also the first study to analyze focus groups through polar coordinates. The observations made suggest that migrant families of Pakistani origin, especially mothers, dedicate resources and effort to the maintenance of values and practices from their country of origin, and their transmission to the following generations. New technologies, which allow constant communication with the relatives in the country of origin, the desire to return and the absence of spaces for interaction between native and migrated families, facilitate the maintenance of the upbringing model of the homeland and resistance to change. Even so, certain contextual conditions deriving from migration, such as a structure of coexistence limited to the nuclear family and access to the educational system and the health system promote acculturation processes and favor adaptation to the new context. Although our study was carried out in a specific geographical context, Catalonia, we believe that our conclusions can be extended to Pakistani mothers who have migrated to other highly industrialized and economically developed regions in Europe. The data provided by this study can constitute a basis for future research on migrant motherhood and are likely to be relevant for professionals working with migrant mothers of Pakistani origin. The activation and deactivation relationships established through polar coordinate analysis can help identify the most suitable questions for obtaining a certain type of information from participants in focus groups or discussion groups, or through interviews or questionnaires in both research and professional contexts.

### Limitations and future lines of research

Certain limitations must be considered. The main limitation refers to the representativeness of the sample participants (Denton, 2007). Recruitment through female leaders from the community and social organizations may include a bias in the participants’ profile. Those most predisposed to participating in gathering and mutual learning spaces with other women are likely to have been chosen. These women may be more predisposed to share experiences of maternity.

Second, the observers who coded the data were not blind to the objectives of the study. Future research may consider blinding observers to avoid bias.

Given the qualitative nature of the data, other quantitative analysis techniques could also be used. For instance, the detection of T-Patterns has proved to be highly applicable in recent years, both in direct observation (Pic et al., 2018, 2020) and in indirect observation (Arias-Pujol and Anguera, 2020; Blanchet et al., 2005). Since this technique aims to detect regularities over time, and since in indirect observation counting the duration of each uttered phrase would not be feasible, conventional units should be used instead of temporal ones.

Finally, we must note the language barrier. Even though the researcher acted as a moderator, guided the dialogue between the participants, gave the floor and encouraged equitable participation, the intervention was limited by the need to have the mediation of a Spanish-Urdu interpreter, with the aim of facilitating communication between the parties. In this sense, we prioritized communication in Urdu, the vehicular language of women, to offer a space for natural and fluid conversation between them.

New investigations will be able to examine more exhaustively some aspects derived from data, such as interactions between other focal and conditioned behaviors, or from other issues such as the influence of new technologies on the perception of loneliness in maternity in migrant mothers, the characteristics that define a culturally sensitive parenting space, or the communication channels mothers use to find out about the resources that the area offers. Likewise, it will be necessary to include the voice and experience of fathers as figures involved in bonding with and raising the children.

## Data Availability

The raw data supporting the conclusions of this article will be made available by the authors, without undue reservation.

## Footnotes

^1^ https://www.mangold-international.com/en/products/software/gseq.html

^2^ https://jairodmed.shinyapps.io/HOISAN_to_R_2022/
