# Supplementary material for: Being a Pakistani mother in Catalonia: a mixed methods study
Source: Front Psychol. 2024 Nov 26;15:1386029. doi: 10.3389/fpsyg.2024.1386029 (PMC11628259; doi:10.3389/fpsyg.2024.1386029)
Supplement: Supplementary file 1 [file Table_1.docx]

Supplementary Material

**Supplementary Table 1.** *Demographic characteristics of the sample of participants in focus groups 1, 2 and 3.*

|  | | Gender | | Age | Profession | Origin (rural or urban in Pakistan) | | Education level | Socioeconomic level | | | Number of children | | Children's age | | Years of residence in Catalonia | |
| --- | --- | --- | --- | --- | --- | --- | --- | --- | --- | --- | --- | --- | --- | --- | --- | --- | --- |
| focus group 1 | woman | | 40 | | social worker | | urban | College students | | from 746-1,312 net/month | 5 | | 5a | | 1a | |  |
|  | woman | | 34 | | housewife | | urban | Primary studies not completed | | from 746-1,312 net/month | 3 | | 12y,9y,3m | | 1a | |  |
|  | woman | | 38 | | teacher | | urban | Upper secondary education | | from 746-1,312 net/month | 2 | | 6y, 8m | | 4a | |  |
|  | woman | | 31 | | housewife | | rural | Secondary education | | from 1,3013 to 1,602 net/month | 3 | | 8a,5a,24m | | 2a | |  |
|  | woman | | 34 | | assistant association | | urban | Postgrad studies | | from 746-1,312 net/month | 1 | | 36m | | 2a | |  |
|  | woman | | 30 | | housewife | | urban | Secundary education | | From 746-1.312 netos/mes | 2 | | 4a | | 3a | |  |
|  | woman | | 31 | | housewife | | rural | Primary studies not completed | | from 1,3013 to 1,602 net/month | 2 | | 5y, 9m | | 1m | |  |
| focus group 2 | woman | | 49 | | housewife | | rural | University studies | | from 1,3013 to 1,602 net/month | 3 | | 18y, 14y, 37m | | 3a | |  |
|  | woman | | 34 | | housewife | | urban | University studies | | from 746-1,312 net/month | 1 | | 6m | | 4a | |  |
|  | woman | | 31 | | teacher | | urban | University studies | | from 746-1,312 net/month | 1 | | 12m | | 4a | |  |
|  | woman | | 35 | | housewife | | rural | University studies | | from 1,603 to 2,145 net/month | 4 | | 9y, 6y, 5y, 35m | | 5a | |  |
|  | woman | | 35 | | teacher | | urban | Postgrad studies | | from 1,3013 to 1,602 net/month | 2 | | 6y, 4m | | 4a | |  |
|  | woman | | 36 | | housewife | | rural | Secondary education | | from 1,603 to 2,145 net/month | 2 | | 6a, 12m | | 3a | |  |
|  | woman | | 37 | | housewife | | urban | University studies | | Less than 745 net/month | 1 | | 36m | | 2a | |  |
| focus group 3 | woman | | 36 | | housewife | | urban | University studies | | Less than 745 net/month | 2 | | 7a, 36m | | 4a | |  |
|  | woman | | 35 | | housewife | | rural | Postgrad studies | | from 746-1,312 net/month | 4 | | 9y, 7y, 39m, 12m | | 5a | |  |
|  | woman | | 29 | | housewife | | urban | Upper secondary education | | from 746-1,312 net/month | 2 | | 4y, 24m | | 5a | |  |
|  | woman | | 35 | | housewife | | urban | Secondary education | | from 746-1,312 net/month | 3 | | 9a, 5a, 7a, 24m | | 5a | |  |
|  | woman | | 34 | | housewife | | urban | Secondary education | | from 746-1,312 net/month | 1 | | 36m | | 4a | |  |
|  | woman | | 40 | | housewife | | urban | Secondary education | | from 746-1,312 net/month | 3 | | 11y, 6y, 36m | | 3a | |  |
