# Supplementary material for: Being a Pakistani mother in Catalonia: a mixed methods study
Source: Front Psychol. 2024 Nov 26;15:1386029. doi: 10.3389/fpsyg.2024.1386029 (PMC11628259; doi:10.3389/fpsyg.2024.1386029)
Supplement: Supplementary file 2 [file Table_2.docx]

**Supplementary Table 2.** *Indirect observation instrument built with focus group narratives. The codes assigned to each dimension and sub-dimension are listed.*

| DIMENSION | CODE | DEFINITION | EXAMPLE |
| --- | --- | --- | --- |
| CA CHANGES AFTER MATERNITY | CA1 Organization | It refers to the changes that occur after maternity in timing, priorities, and new tasks that are added to the usual responsibilities of caring for the family and home. | *Changes in routine, we get up at night, do not sleep, cook* |
|  | CA21 Changes in the focus of conversation | It refers to changes in the focus or topics of conversation. | *When we talk as a family, the conversations revolve around the children. Conversations change.* |
|  | CA22 Consolidation of the mother’s position in the family | It refers to the changes that occur after maternity in the position that the mother occupies, the respect she receives and her consolidation as a member of the family-in-law. | *When your child is born the relationship is stronger.* |
|  | CA3 Tension in the distribution of time between husband and child | It refers to the changes that occur after maternity in terms of conflicts in the couple relationship caused by prioritizing care and spending time caring for children and raising them. | *My husband tells me that after having our son, I have forgotten him.* |
|  | CA41 Competencies | It refers to new learning, skills and resources that mothers acquire after maternity and derived from it. | *When a woman works for something or someone she gets tired, but for her children she never gets tired, quite the opposite.* |
|  | CA42 Loss of freedom | It refers to the loss of freedom of the mother and the loss of personal time. | *Before you have a freer mind.* |
| SE SENSE AND MEANING OF MOTHERHOOD | SE111 Criticism of the extended family towards non-mothers | It refers to comments and criticism that women receive from the  Relatives when they do not get pregnant after marriage. | *After you get married, if you don’t have children, your husband’s family starts to worry a lot. They suspect.* |
|  | SE112 Extended family support for non-mothers | It refers to comments about the support that women receive from the relatives when they do not become pregnant after marriage. | *My mother-in-law used to tell me that if there was a child in our luck, it would come from me,* |
|  | SE12 Social pressure on non-mothers | It refers to the social pressure exerted by the community/society when women do not become pregnant after marriage. | *but society or distant relatives spoke and I felt bad.* |
|  | SE21 Responsibility | It refers to the perception of maternity and cares as the responsibility of women. | *She has all the responsibility.* |
|  | SE22 Life objective | It refers to the perception of motherhood as a life goal. | *If there are no children, you are incomplete.* |
|  | SE23 Divine mandate | It refers to the perception of motherhood as a divine mandate. | *They are children. If Allah has given them to us we have to raise them.* |
| MO PARENTING MODEL | MO11 Support of the extended family in bringing up their children | It refers to the support offered by the family-in-law in raising and caring for children. | *I had my first daughter in Pakistan and another here, and I had the help of my sisters-in-law and mother-in-law.* |
|  | MO12 Responsibilities with extended family make parenting difficult | It refers to comments on the responsibility and tasks assumed by mothers in the care of the family-in-law hat make parenting difficult. | *When you are with your in-laws you have more responsibilities or when you are at your parent’s house.* |
|  | MO2111 Present family values | It refers to cultural values that are transmitted in the family. | *Here they give more attention to religion.* |
|  | MO2112 Loss of values after migration | It refers to the loss of cultural values in children after migration. | *They think that only parents and no one else should be respected.* |
|  | MO212 Fear of setting limits | It refers to descriptive aspects of the parenting model in terms of the need to establish friendly relations with the children, not to be excessively strict, as a manifestation of the fear of losing them. | *They have to be like friends, you can’t be hard on them or else they’ll get out of hand.* |
|  | MO22 Difficulties due to the loss of extended family in support for bringing up the children | It refers to limiting situations derived from living only with the nuclear family. Comments are included regarding the loss of family-in-law support. | *You have to do everything alone, raise the child, and take care of the family.* |
|  | MO31 Personal autonomy | It refers to the teaching of skills related to the autonomy of children. | *Little by little when they grow up we will give them small responsibilities, bringing something from the kitchen for example, and thus they will grow disciplined.* |
|  | MO32 Religion | It refers to the teaching of religious values and practices to the children of the family. | *In the first three years, the only thing I have been able to teach him is religious education.* |
|  | MO411 Transmission of cultural values, practices and attitudes | It refers to the teaching and transmission of culturally determined practices, values and attitudes. | *The child learns a lot from his mother. The first school is the mother.* |
|  | MO412 Responsible for care and upbringing | It refers to the role of the mother as responsible for the care and upbringing of her children. | *The mothers are with them all day.* |
|  | MO413 Surveillance | It refers to the need to maintain constant vigilance to prevent children from losing cultural and religious values or acquiring bad habits. | *Watch them more and give them more attention.* |
|  | MO414 Support in studies | It refers to the role of the mother as responsible for the studies and educational follow-up of the children. | *When they return from school we have to tell them to sit down, to study.* |
|  | MO415 Emotional support and link | It refers to the role of the mother as an emotional and bonding support. | *The mother bears the child for life.* |
|  | MO416 Husband care | It refers to the need to offer attention and care to the husband. | *You also have to dedicate time to your husbands, as I told you.* |
|  | MO421 Family economy | It refers to the father’s role as the economic breadwinner of the family. | *The man also has a reason to work.* |
|  | MO422 Indulge children | It refers to the presence and role of the father as a figure that consents to the whims of the children. | *I am sometimes stricter with her but he is like a friend.* |
|  | MO423 Bond and emotional support | It refers to the role of the father as a figure of emotional support and link for the children. | *Husbands care more and worry about the children.* |
|  | MO424 Pick up children from school | It refers to the tasks assigned to fathers in raising children: picking up their children from school. | *Sometimes he calls me and asks me to pick her up, but usually, he does it.* |
|  | MO425 Tasks requiring linguistic competence | It refers to the tasks assigned to fathers in raising children in spaces in which linguistic competence is required (medical visits, school visits, administration, etc.) | *If you can’t due to a lack of language, your husband can go.* |
|  | MO426 Little or no involvement in parenting | It refers to comments about little or no involvement of the father in upbringing. | *My husband does nothing with his daughter.* |
|  | MO427 Recreational activities | It refers to tasks assigned to fathers in raising children: playful activities with their children. | *When he returns he eats with the children, he tells them stories.* |
|  | MO428 School monitoring | It refers to the tasks assigned to fathers in parenting: school follow-up. | *My husband asks the elders about the routine at school, and what they have done.* |
|  | MO5 Culturally determined practices | It refers to culturally determined practices in the family. | *They have a religious upbringing and practice Ramadan.* |
| NE BASIC INDIVIDUAL NEEDS OF FAMILY MEMBERS | NE11 Mothers’ emotional well-being | It refers to the need for emotional well-being that is felt and expressed by mothers in parenting. | *The basic thing is that the mother has had a good childhood so that her son also has it.* |
|  | NE12 Family support | It refers to the need for support and relatives support felt and expressed by mothers. | *We remember our mothers and mothers-in-law.* |
|  | NE13 Physical well-being of the mother | It refers to the need for physical well-being in the mother to be able to exercise parenting. | *The second important thing is that the mother is in good health, if she is healthy, she will be able to do everything better.* |
|  | NE21 Link | It refers to the basic need for bonding of children with their mothers/fathers or to needs that are expressed as bond generators. | *If there is no bond there is no difference between a mother and a stranger.* |
|  | NE22 Time and space | It refers to the basic need to spend quality time children with their parents. | *Here I have been able to dedicate more time to them and the relationship is better.* |
|  | NE23 Home environment | It refers to the basic need to live in a home free of family conflicts. | *The husband and wife also have to care for each other and the children, seeing this, they are also happy.* |
|  | NE24 Game | It refers to the basic need for play in the child. | *What do they do in the first three years, play and some religious education?* |
| AC CULTURATION | AC111 Professional indication produces changes in culturally determined practices | It refers to changes in the upbringing in cultural aspects that occur after professional indications. | *If we have to change something, we change it and pay attention.* |
|  | AC112 Cultural practice does not change after professional indications | It refers to culturally determined practices that are maintained in parenting despite the professional indication of change. | *Yes, the doctors tell us, but when we get home we still put it on.* |
|  | AC121 No relationship | It refers to the lack of relationship or connection with Catalan families. | *We don’t know how to talk and we can’t relate* |
|  | AC122 Positive assessment of childrearing practices in Catalonia | It refers to aspects of upbringing in Catalan families that are valued positively. | *They also have very marked schedules and we don’t.* |
|  | AC123 Negative assessment of childrearing practices in Catalonia | It refers to aspects of upbringing in Catalan families that are valued negatively. | *They buy bread, we have to prepare the dough and make the bread.* |
|  | AC124 Negative assessment of own parenting practices | It refers to criticisms of aspects of the parenting model itself. | *If a Catalan mother says no to her son, she remains firm in her decision, not us, if she starts to cry a little, we give in.* |
|  | AC211 More dedication of time and/or a better link | It refers to the possibility of spending more time with children in Catalonia and its impact on the mother-child bond. | *Here I have been able to dedicate more time to them and the relationship is better.* |
|  | AC212 Better care from public services and resources | It refers to better care and opportunities for public services and resources in Catalonia. | *The facilities provided by the system, also in health, there are none.* |
|  | AC213 It improves the transmission of cultural values and practices | It refers to the possibility of carrying out a greater/better transmission of values and cultural practices of origin in Catalonia. | *Here they give more attention to religion.* |
|  | AC221 Increased responsibility and increased tasks | It refers to the overload of tasks suffered by mothers in Catalonia. | *Here you have to do it all.* |
|  | AC222 Greater difficulty in work-life balance | It refers to the difficulties of reconciliation when mothers work outside the home. | *Here, from morning to night, I work.* |
|  | AC223 Cultural and linguistic differences hinder social inclusion | It refers to the difficulties generated by linguistic and cultural differences in social inclusion. | *The main problem for us is clothing.* |
|  | AC224 Difficulty accompanying and guiding children in their studies | It refers to the difficulties expressed by mothers in not being able to accompany their children in their studies due to language barriers and cultural differences. | *Of course, teaching children is very complicated.* |
|  | AC225 Differences inside/outside the home cause confusion in children | It refers to the confusion that living between two different cultures and languages generates in children. | *They don’t understand things from outside and we can’t explain it to them. What they learn at home they cannot apply outside.* |
|  | AC231 Family-in-law link | It refers to the greater bond that is established with the family-in-law when it lives together. | *Children spent time with their grandparents or uncles.* |
|  | AC232 Family-in-law support in caring for children and mother | It refers to the support offered by the family-in-law in raising and caring for children as a positive aspect of living in Pakistan. | *There you only have to give birth and the others take care of them.* |
|  | AC233 Ease of work-life balance | It refers to the facilities that mothers in Pakistan find to reconcile their work and family life. | *In Pakistan, if you are not living with your family, you can easily find someone and have a woman by your side to help you.* |
|  | AC234 More options to occupy free time | It refers to a greater offer of activities and/or people who offer entertainment in Pakistan. | *We go to relatives’ houses, or they come to our houses. Kids are always entertained.* |
|  | AC235 Better education system and/or easier way to support children in their studies | Refers to comments about better learning/educational levels in Pakistan and/or better possibility to track children’s studies. | *The eldest went to school in Pakistan and was able to help him.* |
|  | AC236To share cultural values and practices | The possibility of sharing cultural values and practices with the society in which you live is perceived as a positive aspect of living in Pakistan. | *Nothing happens in Pakistan because the society has the same culture.* |
|  | AC241 Other responsibilities make it difficult to dedicate time to parenting | The responsibilities and care tasks of the family-in-law make it difficult to dedicate time to parenting. | *Positive, I think that in Pakistan we cannot spend a lot of time with them because there is a lot of family and you have to do a lot of things.* |
|  | AC242 Excessive educational pressure | It refers to comments related to the perception of increased educational pressure on children as a source of stress in Pakistan. | *There you only look at the number of the mark they get as if the world were going to end.* |
|  | AC243 Non-social equity | It refers to social inequality and the lack of resources and opportunities for lower-income families in Pakistan. | *The poor kids who get good grades are ahead of the rich because they don’t have contacts in the system.* |
|  | AC244 Limitations of public services and resources | It refers to the negative aspects of public services and resources in Pakistan. | *In Pakistan this does not exist. Education is not promoted in Pakistan.* |
|  | AC245 Difficulty controlling habits and behavior of children | It refers to less control of children, their companions and behavior in Pakistan. | *In Pakistan if you have bad company... and here the same.* |
|  | AC3 Desire to return | It refers to contextual and emotional aspects that motivate the desire to return | *My daughter came from Pakistan and stopped seeing her family. Now when she goes to their house (she points to one of the participants) she calls her and repeats “aunt Roma, aunt Roma”. She asks us to go back to Pakistan because she doesn’t have her family here.* |
|  | AC4 Concerns about loss of cultural/religious values and/or acquisition of practices of the host society | It refers to manifestations, verbal or behavioral, that show concern for the loss of cultural and/or religious values of origin and/or the acquisition of values or practices of the host society. | *Our religion cannot be left for nothing.* |
| RE SUPPORT NETWORK | RE111 Information between equals | It refers to the support received from other women of Pakistani origin who offer information on parenting or mother/childcare. Women belonging to the extended family are not included. | *Discussing things among everyone helps a lot, what you don’t know you can learn from another.* |
|  | RE112 Caring between equals | It refers to the support received from other women of Pakistani origin who were involved in caring for mothers and/or children during the quarantine period. Women belonging to the extended family are not included. | *We help each other. I have had two children here and she (points to one of the participants) has helped me a lot. Here we help each other even more because we know there is no family.* |
|  | RE211 Information support from the mother’s family members in Pakistan | It refers to the informative support and resolution of doubts received by the mother’s family members in Pakistan. | *Yes, in Pakistan we can communicate with our extended family, with sisters-in-law, my mother, my mother-in-law… with experienced relatives.* |
|  | RE221 Activities of daily living of family-in-law in Catalonia | It refers to the support in activities of daily living that mothers receive from members of the family-in-law residing in Catalonia. | *I leave one with his grandmother and I take another to school. I have this support from my family but she doesn’t.* |
|  | RE222 Family-in-law information in Catalonia | It refers to the support that mothers receive in resolving doubts or receiving information from members of the family-in-law residing in Catalonia. | *I ask my sisters-in-law who are here and have children.* |
|  | RE311 Opportunities offered by the health system | It refers to opportunities perceived by mothers about the resources offered by health services in Catalonia. | *Here there is help from doctors if you need psychological help, there are psychologists.* |
|  | RE321 Opportunities of the educational system | It refers to the opportunities offered by the educational system in the care and education of the children of families. Comments are included regarding the support that the family receives in general. | *If a child has a mental problem or if he needs psychological attention, they monitor him.* |
|  | RE322 Limitations of the educational system | It refers to the limitations that they perceive in the Catalan educational system in the education of the children of the families. | *We have just arrived and we see that my son’s level of studies is very backward.* |
|  | RE323 Concern for the loss of languages of origin | It refers to the perception of negative aspects of the educational system in Catalonia that are related to the loss of the languages of origin (Urdu and English). | *If they don’t know English or Urdu, they won’t be able to go to work anywhere, they will only have the option of being here.* |
|  | RE3311 Barriers to participation | It refers to the appearance of barriers to access and participate in formal spaces for parenting and maternity support or other learning spaces when there is a perceived need or interest in participating. | *She has not had a problem because she has a family to leave her children with. But our husbands tell us that it is not right to leave our children to someone.* |
|  | RE3312 Opportunities to participate | It refers to the opportunities offered by participation in formal spaces for parenting and maternity support or other learning spaces when there is a perceived need or interest in participating. | *It is necessary to have this type of space to resolve doubts. Even if it’s once every three months. Today they met, and when we are in a group some reacted to this issue, they did not know about it before. There has to be something to be able to share, to create a community.* |
|  | RE332 No need/interest in participating | It refers to the lack of need or interest in participating in formal support spaces for parenting. | *It is natural, when you are a mother, nature teaches you everything.* |
|  | RE34 Other services that offer information and support | It refers to informal resources that offer information on raising and caring for children. | *The Internet helps me a lot. Mom Google. When I have any doubts, I search. I made an email account and signed up for pages and they sent me notifications with information. Since I have no one to ask, Google helps me.* |
